# The Effects of Matriptase Inhibition on the Inflammatory and Redox Homeostasis of Chicken Hepatic Cell Culture Models

**DOI:** 10.3390/biomedicines9050450

**Published:** 2021-04-21

**Authors:** Réka Fanni Barna, Máté Mackei, Erzsébet Pászti-Gere, Zsuzsanna Neogrády, Ákos Jerzsele, Gábor Mátis

**Affiliations:** 1 Division of Biochemistry, Department of Physiology and Biochemistry, University of Veterinary Medicine, 1078 Budapest, Hungary; mackei.mate@univet.hu (M.M.); neogrady.zsuzsanna@univet.hu (Z.N.); matis.gabor@univet.hu (G.M.); 2 Department of Pharmacology and Toxicology, University of Veterinary Medicine Budapest, 1078 Budapest, Hungary; gere.erzsebet@univet.hu (E.P.-G.); jerzsele.akos@univet.hu (Á.J.)

**Keywords:** serine proteases, proinflammatory cytokines, oxidative stress, poultry, liver

## Abstract

The function of the transmembrane serine protease matriptase is well described in mammals, but it has not been elucidated in avian species yet. Hence, the aim of the present study was to assess the effects of the 3-amidinophenylalanine (3-AphA)-type matriptase inhibitors MI432 and MI460 on the inflammatory and oxidative state of chicken primary hepatocyte mono-cultures and hepatocyte–nonparenchymal cell co-cultures, the latter serving as a proper model of hepatic inflammation in birds. Cell cultures were exposed to MI432 and MI460 for 4 and 24 h at 10, 25, and 50 µM concentrations, and thereafter the cellular metabolic activity, extracellular interleukin (IL-)6, IL-8, H_2_O_2_ and malondialdehyde concentrations were monitored. Both inhibitors caused a transient moderate reduction in the metabolic activity following 4 h exposure, which was restored after 24 h, reflecting the fast hepatic adaptation potential to matriptase inhibitor administration. Furthermore, MI432 triggered an intense elevation in the cellular proinflammatory IL-6 and IL-8 production after both incubation times in all concentrations, which was not coupled to enhanced oxidative stress and lipid peroxidation based on unchanged H_2_O_2_ production, malondialdehyde levels and glutathione peroxidase activity. These data suggest that physiological matriptase activities might have a key function in retaining the metabolic and inflammatory homeostasis of the liver in chicken, without being a major modulator of the hepatocellular redox state.

## 1. Introduction

Matriptase belongs to the type II transmembrane serine proteases (TTSPs), which have a key role in several physiological and pathological processes [1]. Concerning the various isoenzymes, matriptase-1 is widely expressed in all types of epithelial tissues [2,3], while matriptase-2 is mostly localized in the liver [4]. Matriptase is required for postnatal survival to establish and maintain barrier function of the epithelium [5], while improperly regulated matriptase may be involved in a number of pathological conditions, such as tumorigenesis [6], inflammation [7], virus-cell fusion [8,9], or even impaired iron homeostasis [10,11,12].

The inhibition of matriptase with specific inhibitors, such as the recently developed 3-amidinophenylalanine (3-APhA) derivatives, may represent a new, reliable therapeutic approach for numerous diseases related to matriptase deregulation. For instance, based on in vitro [13,14] or in vivo [15] studies, the application of 3-APhA inhibitors can effectively reduce the growth and invasion potential of tumor cells [13,14], and may possess antiviral [8], antirheumatic [16] or hepcidin-lowering effects [17]. The key role of matriptase in retaining gut-barrier integrity was confirmed on the IPEC-J2 porcine nontransformed small intestinal epithelial cell line, where the inhibitors MI432 and MI460 decreased the transepithelial electrical resistance and increased paracellular transport mechanisms [18]. A short-term exposure of swine-derived hepatic cell culture models to MI441 caused elevated cellular reactive oxygen species (ROS) release coupled to reduced hepcidin expression, while MI460 significantly increased hepcidin secretion [17]. Notwithstanding that matriptase can initiate the protease-activated receptor (PAR)-2-mediated inflammatory pathway [19], the hepatocellular production of the proinflammatory cytokines IL-6 and IL-8 was not affected by the applied 3-APhA type inhibitors on the same cell cultures [17].

There are only few studies investigating matriptase in avian species. Matriptase was mainly detected in mammalian cells; however, its ortholog [20] and matriptase-3 [21] were found in the chicken genome as well. The matriptase-driven proteolytic activation of H9N2 influenza virus was examined in chicken embryo kidney cells. Moreover, the cleavage of hemagglutinin was inhibited by the 3-APhA inhibitor MI462 at 50 µM concentration [8].

As the regulatory action of matriptase in birds has not been elucidated yet, the major aim of the present study was to assess the function of this membrane-anchored serine protease in the liver of chickens by applying the 3-APhA type inhibitors MI432 and MI460 on chicken primary hepatocyte mono- and hepatocyte–nonparenchymal (NP) cell co-cultures. These cell systems provide proper tools for biomedical research of the avian liver, the co-culture serving as a model of the hepatic inflammation. The inhibitors MI432 and MI460 were selected because they have already been effective to inhibit matriptase without harmful cytotoxic effects on mammalian cells [17,18,22]. To fulfill the abovementioned goals, the cellular metabolic activity and oxidative status, as well as the production of proinflammatory cytokines, were monitored, reflecting the role of matriptase in regulating redox and inflammatory homeostasis of the liver.

## 2. Materials and Methods

### 2.1. Cell Isolation and Culturing

Liver cells were isolated and cell cultures were prepared as described in a previous study of our research group [23], using three-week-old male Ross-308 type broiler chickens, obtained from Gallus Ltd. (Devecser, Hungary). Cell isolation was carried out in accordance with national and EU laws and institutional guidelines, also confirmed by the Local Animal Welfare Committee of the University of Veterinary Medicine, Budapest. Experimental procedures were officially approved by the Government Office of Budapest, Food Chain Safety, Plant Protection, and Soil Conservation Directorate, Budapest, Hungary (number of permission: PEI/001/1430-4/2015; approval date: 27 April 2015). All chemicals were purchased from Merck KGaA (Darmstadt, Germany) except when otherwise specified.

Briefly, a multistep in situ perfusion of the liver was performed via the gastropancreaticoduodenal vein, applying 150 mL ethylene glycol bis (2-aminoethyl ether) tetraacetic acid (EGTA, 0.5 mM) containing Hanks’ Balanced Salt Solution (HBSS) with 0.035% NaHCO_3_, 150 mL EGTA-free HBSS, and finally, 100 mL HBSS buffer supplemented with 100 mg collagenase type IV (Nordmark, Uetersen, Germany), 7 mM CaCl_2_, and 7 mM MgCl_2_ to disintegrate hepatic cells. After disrupting the capsule of the liver, the gained cells were suspended in 2.5% bovine serum albumin (BSA) containing HBSS and were filtered through three layers of sterile gauze, followed by a 45 min incubation on ice. Thereafter, hepatocytes and NP cells were isolated by a multistep differential centrifugation as previously described [23]. Both cell fractions were resuspended in Williams’ Medium E, supplemented with 0.22% NaHCO_3_, 50 mg/L gentamycin, 2 mM glutamine, 4 µg/L dexamethasone, 20 IU/L insulin, and 5% foetal bovine serum (FBS). The viability of hepatocytes and NP cells was examined by the trypan blue exclusion test, and cell suspensions were diluted after cell counting in a Bürker chamber to the appropriate seeding concentrations (hepatocyte mono-cultures: 10^6^ cells/mL; co-cultures: 8.5 × 10^5^ cells/mL hepatocytes, 1.5 × 10^5^ cells/mL NP cells). Both hepatocyte and NP cell enriched fractions have been previously characterized by flow cytometry and immunofluorescent detection of specific markers for hepatocytes and macrophages [23].

**Figure 1:**
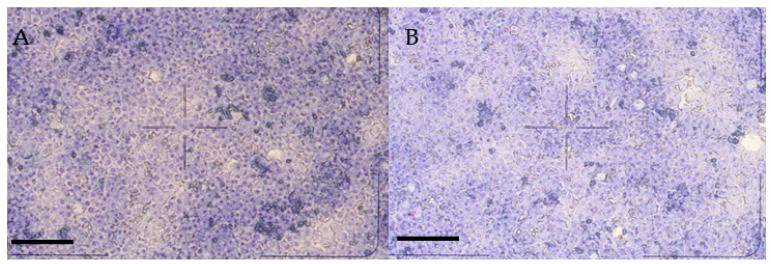
Giemsa staining of hepatocyte mono-cultures (**A**) and hepatocyte–nonparenchymal cell co-cultures (**B**) after 48 h culturing (20× magnification, bar = 100 µm).

Cells were seeded on 6-well (to monitor extracellular H_2_O_2_, interleukin (IL-)6, IL-8, and malondialdehyde (MDA) concentrations and intracellular glutathione peroxidase (GPx) activities) or 96-well (to assess cellular metabolic activity) plates (Greiner Bio-One, Frickenhausen, Germany), previously coated with collagen type I (10 µg/cm^2^). To establish the co-cultures, NP cells were seeded first, and following their adherence in 20 min, hepatocytes were added in the cell ratio of 6:1 (hepatocyte to NP cells). Hepatocyte mono-cultures were also prepared by seeding only the hepatocyte-enriched fraction. The seeding volume was 1.5 mL/well on 6-well plates and 100 µL/well on 96-well plates. All cell cultures were incubated at 38.5 °C in humid atmosphere with 5% CO_2_. Culture media were changed 4 h following seeding; hepatocyte mono-cultures (Figure 1A) and hepatocyte–NP cell co-cultures (Figure 1B) formed confluent monolayers after 24 h culturing [23].

### 2.2. Treatments and Samplings

**Figure 2:**
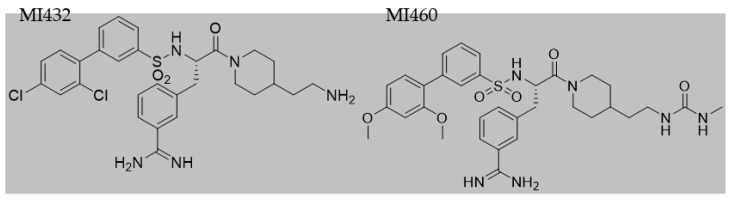
Structures of the used matriptase inhibitors.

After 24 h incubation, culture media were removed and cultures were exposed to the inhibitors MI432 and MI460 for 4 and 24 h at 10, 25, and 50 µM concentrations. The structures (Figure 2) and inhibitory constant (Ki) values (Table 1) of the applied MI432 and MI460 inhibitors were already published by Hammami et al. [24]. All 3-APhA inhibitors were kindly provided by the Institute of Pharmaceutical Chemistry, Faculty of Pharmacy, Philipps University, Marburg, Germany.

Culture media of 6-well plates were collected directly after the incubation time to assess extracellular H_2_O_2_, IL-6, IL-8, and MDA concentrations, while cells were lysed in Mammalian Protein Extraction Reagent (M-PER) buffer supplemented with 1% Halt Protease Inhibitor Cocktail and 1% ethylene diamine tetraacetic acid (EDTA) (Thermo Fisher Scientific, Waltham, MA, USA). To standardize the results, total protein concentrations of cell lysates were measured with Pierce Bicinchoninic Acid (BCA) Protein Assay (Thermo Fisher Scientific, Waltham, MA, USA), using BSA as a standard, following the manufacturer’s description. All samples were stored at −80 °C until further processing.

### 2.3. Measurements of Cellular Metabolic Activity, IL-6, IL-8, H_2_O_2_ and MDA Concentrations and GPx Activity

Following 4 and 24 h inhibitor treatment, the metabolic activity of cells on 96-well plates was assessed by the Cell Counting Kit-8 (CCK-8) assay (Merck KGaA, Darmstadt, Germany) according to the manufacturer’s instructions, to detect the amount of NADH+H^+^ generated in the catabolic pathways. Cells were incubated with 10 µL CCK-8 reagent added to 100 µL fresh Williams’ Medium E, and the absorbance was measured at 450 nm with a Multiskan GO 3.2 reader (Thermo Fisher Scientific, Waltham, MA, USA) after 2 h incubation at 38.5 °C.

The concentrations of IL-6 and IL-8 from cell-free supernatants were assayed by chicken-specific double-sandwich ELISA methods (MyBioSource, Inc., San Diego, CA, USA), following the manufacturer’s instructions, and measuring absorbances at 450 nm with a Multiskan GO 3.2 reader. The extracellular H_2_O_2_ concentration was monitored with the Amplex Red Hydrogen Peroxide Assay Kit (Thermo Fisher Scientific, Waltham, MA, USA), mixing 50 µL supernatant with 50 µL Amplex Red working solution (composed of 100 µM Amplex Red and 0.2 U/mL horseradish peroxidase), and detecting fluorescence after 30 min incubation at room temperature with a Victor X2 2030 fluorometer (λex = 560 nm; λem = 590 nm).

The MDA as a marker of lipid peroxidation was assayed with the MDA Colorimetric Assay Kit (Merck KGaA, Darmstadt, Germany), adding 300 µL thiobarbituric acid (TBA) stock solution to 100 µL culture media. Blends were incubated at 95 °C for 1 h and thereafter were cooled down on ice for 10 min. Absorbance was measured at 532 nm with a Multiskan GO 3.2 reader (Thermo Fisher Scientific, Waltham, MA, USA).

The GPx activity was assessed from cell lysates using a colorimetric kinetic assay kit (Merck KGaA, Darmstadt, Germany). According to the manufacturer’s instructions, 15 µL cell lysate was added to 455 µL freshly prepared GPx Assay Buffer, 25 µL NADPH Assay Reagent, and 5 µL 30 mM tert-Butyl Hyperoxide solution, the latter serving as the substrate. The absorbance was regularly detected in 10 sec intervals for 1 min at 340 nm. Enzyme activity was determined with the formula provided by the manufacturer.

### 2.4. Statistical Analysis

All treatments were applied in triplicates (except control cells, *n* = 6) on both 6-well and 96-well plates. Results of the measured parameters were standardized to the total protein concentrations of cell lysates. Data were analyzed with one-way ANOVA and Tukey’s post hoc test and Spearman’s test of correlation using the R 3.6.1. software package (Vienna, Austria, 2012; accessed on 15 September 2020). Statistical significance was set at *p* < 0.05; the results are expressed as mean ± SEMs.

## 3. Results

**Figure 3:**
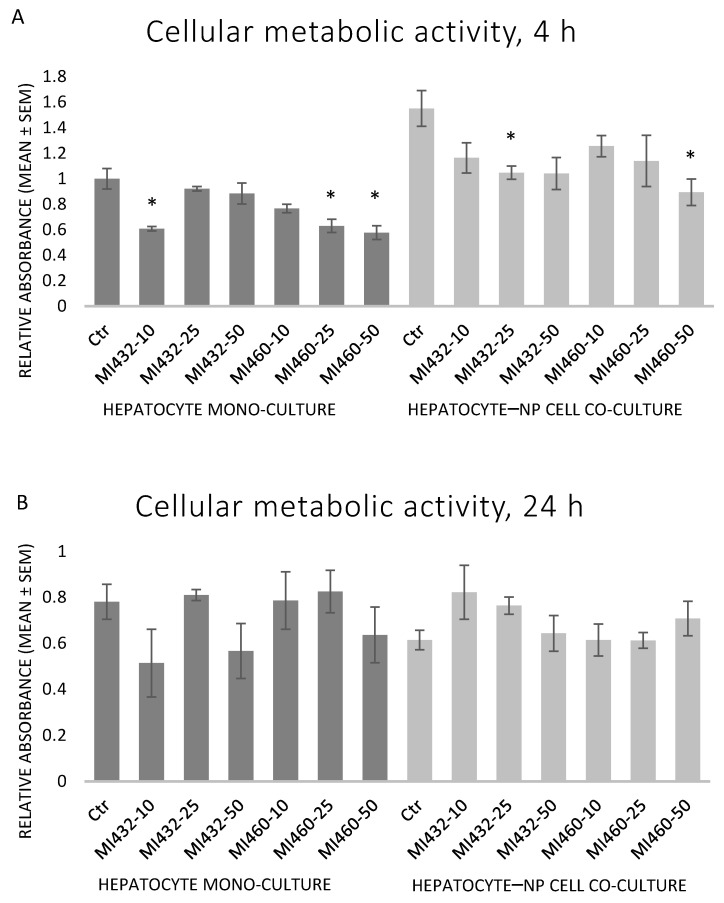
The effects of MI432 and MI460 inhibitors on the metabolic activity of hepatocyte mono-cultures and hepatocyte–NP cell co-cultures after 4 (**A**) and 24 (**B**) h of incubation at 10, 25, and 50 µM concentrations, assessed by a CCK-8 test. *p* values belonging to significant differences: mono-cultures, 4 h: *p*_MI432 10 µM_ = 0.0131; *p*_MI460 25 µM_ = 0.0197; *p*_MI460 50 µM_ = 0.0111; co-cultures, 4 h: *p*_MI432 25 µM_ = 0.0457; *p*_MI460 50 µM_ = 0.0187. Relative absorbance values were calculated by considering the mean absorbance of control hepatocyte mono-cultures at 4 h as 1 (mean ± SEM; *n*_ctr_ = 6, *n*_MI_ = 3/group; * *p* < 0.05).

In the case of the shorter, 4 h treatment, both inhibitors significantly reduced the metabolic activity of the cells in mono- and co-cultures in certain cases, as indicated in Figure 3A. The observed effect was similar in case of exposure to MI432 and MI460 inhibitors in each investigated cell culture model (Figure 3A). In contrast, the longer, 24 h treatment with MI432 or MI460 did not significantly affect metabolic activity of cultured cells (*p* > 0.05 in all cases) (Figure 3B).

**Figure 4:**
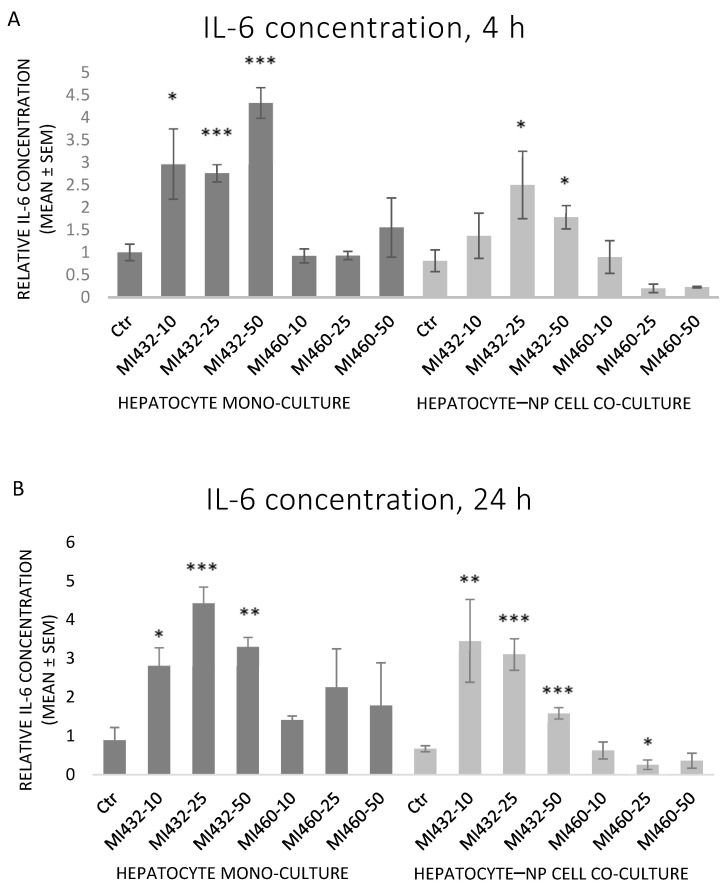
The effects of MI432 and MI460 on the IL-6 production of hepatocyte mono-cultures and hepatocyte–NP cell co-cultures after 4 (**A**) and 24 (**B**) h of treatment at 10, 25, and 50 µM, assessed by the ELISA method. *p* values belonging to significant differences: mono-cultures, 4 h: *p*_MI432 10 µM_ = 0.0115; *p*_MI432 25 µM_ = 0.0006; *p*_MI432 50 µM_ < 0.0001; co-cultures, 4 h: *p*_MI432 25 µM_ = 0.0269; *p*_MI432 50 µM_ = 0.0421; mono-cultures, 24 h: *p*_MI432 10 µM_ = 0.0116; *p*_MI432 25 µM_ = 0.0004; *p*_MI432 50 µM_ = 0.0021; co-cultures, 24 h: *p*_MI432 10 µM_ = 0.0018; *p*_MI432 25 µM_ < 0.0001; *p*_MI432 50 µM_ = 0.0004; *p*_MI460 25 µM_ = 0.0346. Relative concentrations were calculated by considering the mean concentration of control hepatocyte mono-cultures at 4 h as 1 (mean ± SEM; *n*_ctr_ = 6, *n*_MI_ = 3/group; * *p* < 0.05; ** *p* < 0.01; *** *p* < 0.001).

The inhibitors MI432 and MI460 had different effects on the concentration of IL-6 in the supernatants. MI432 significantly increased the concentration of IL-6 for 4 h and 24 h incubation times. This IL-6 elevating effect was more pronounced on mono-cultures for 4 h compared to co-cultures, and the extent was similar for 24 h in both cell models. On the other hand, MI460 did not significantly influence the IL-6 production of cultured cells (except a significant reducing effect on co-culture at 25 μM for 24 h) (Figure 4A,B).

**Figure 5:**
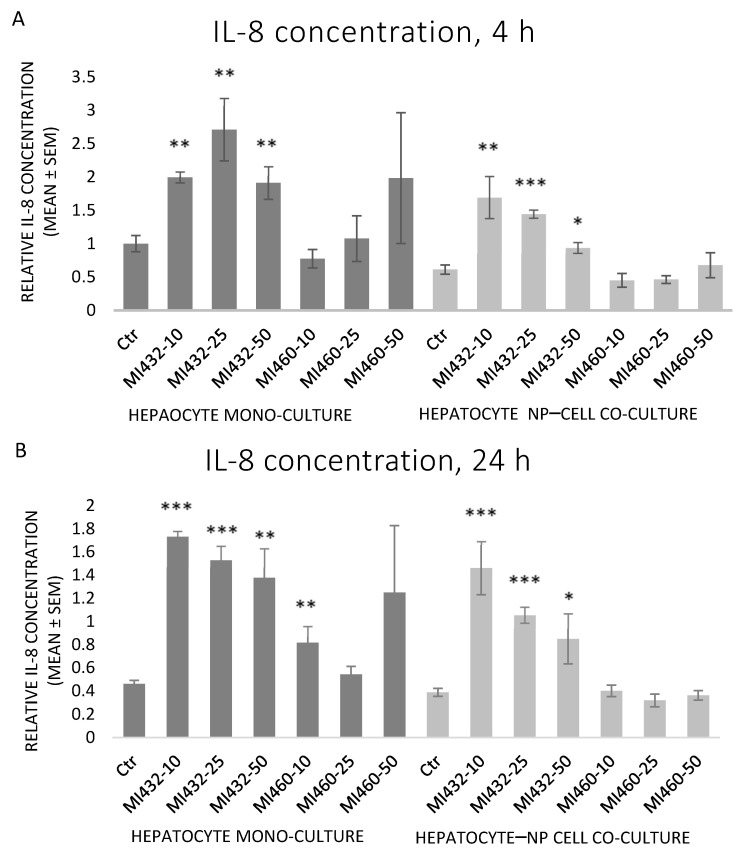
The effects of MI432 and MI460 on the IL-8 production of hepatocyte mono-cultures and hepatocyte–NP cell co-cultures after 4 (**A**) and 24 (**B**) h of treatment at 10, 25, and 50 µM, assessed by the ELISA method. *p* values belonging to significant differences: mono-cultures, 4 h: *p*_MI432 10 µM_ = 0.0011; *p*_MI432 25 µM_ = 0.0019; *p*_MI432 50 µM_ = 0.0068; co-cultures, 4 h: *p*_MI432 10 µM_ = 0.0022; *p*_MI432 25 µM_ = 0.0001; *p*_MI432 50 µM_ = 0.0231; mono-cultures, 24 h: *p*_MI432 10 µM_ < 0.0001; *p*_MI432 25 µM_ < 0.0001; *p*_MI432 50 µM_ = 0.0010; *p*_MI460 10 µM_ = 0.0097; co-cultures, 24 h: *p*_MI432 10 µM_ = 0.0003; *p*_MI432 25 µM_ < 0.0001; *p*_MI432 50 µM_ = 0.0179. Relative concentrations were calculated by considering the mean concentration of control hepatocyte mono-cultures at 4 h as 1 (mean ± SEM; *n*_ctr_ = 6, *n*_MI_ = 3/group; * *p* < 0.05; ** *p* < 0.01; *** *p* < 0.001).

Similar to IL-6, MI432 significantly increased the cellular IL-8 release in each case, on both mono- and co-cultures for 4 and 24 h incubation alike. On the other hand, MI460 did not influence IL-8 levels in mono- or co-cultures at any concentrations for 4 or 24 h (except on mono-cultures at 10 µM for 24 h, which significantly increased it) (Figure 5A,B).

**Figure 6:**
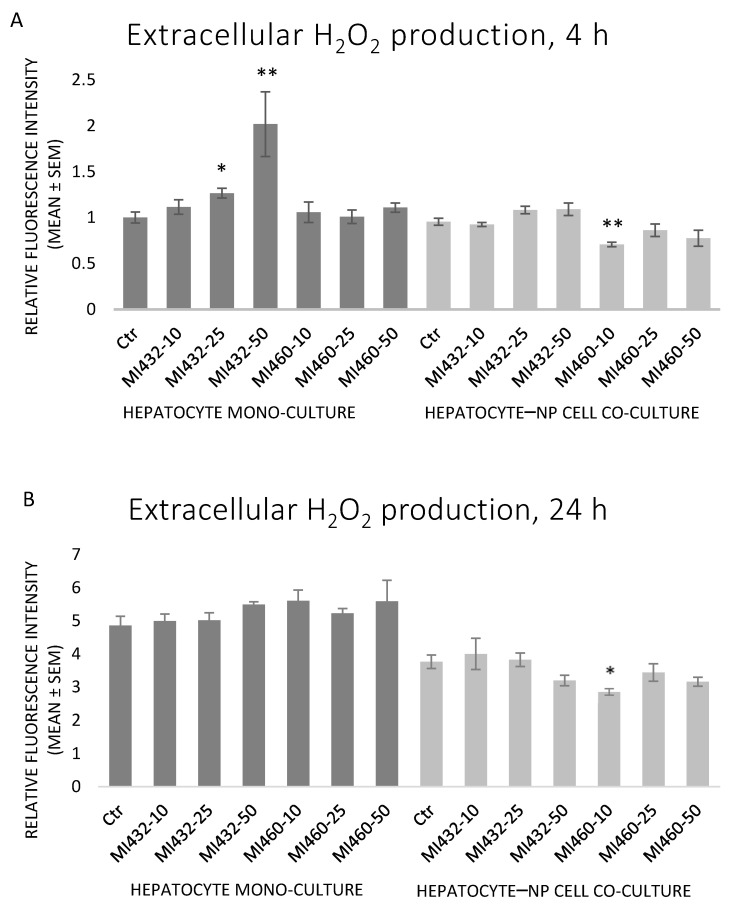
The effects of MI432 and MI460 on the extracellular H_2_O_2_ levels of hepatocyte mono-cultures and hepatocyte–NP cell co-cultures after 4 (**A**) and 24 (**B**) h of treatment at 10, 25, and 50 µM, assessed by the Amplex Red method. *p* values belonging to significant differences: mono-cultures, 4 h: *p*_MI432 25 µM_ = 0.0268; *p*_MI432 50 µM_ = 0.0045; co-cultures, 4 h: *p*_460 10 µM_ = 0.0048; co-cultures, 24 h: *p*_460 10 µM_ = 0.0199. Relative fluorescence intensities were calculated by considering the mean fluorescence intensity of control hepatocyte mono-cultures at 4 h as 1 (mean ± SEM; *n*_ctr_ = 6, *n*_MI_ = 3/group; * *p* < 0.05; ** *p* < 0.01).

There were no significant differences in extracellular H_2_O_2_ levels in most cases when exposing the cells to MI432 or MI460 inhibitors. Exceptionally, MI432 at 25 and 50 μM increased the H_2_O_2_ concentration in culture media after 4 h of incubation on hepatocyte mono-cultures, while a significant decrease was observed when applying MI460 at 10 μM for 4 and 24 h on co-cultures (Figure 6A,B). A significant positive correlation was found between H_2_O_2_ and IL-8 levels on mono-cultures (*p* = 0.019).

**Figure 7:**
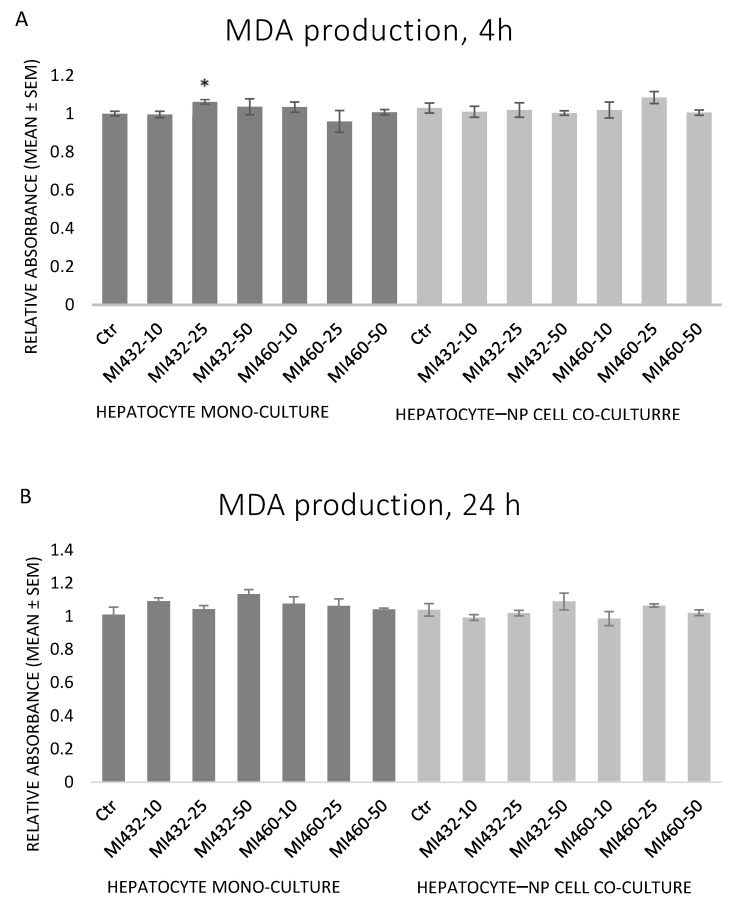
The effects of MI432 and MI460 on the malondialdehyde (MDA) concentration of hepatocyte mono-cultures and hepatocyte–NP cell co-cultures after 4 (**A**) and 24 (**B**) h of treatment at 10, 25, and 50 µM, assessed by a specific colorimetric assay kit. *p* value belonging to the significant difference: mono-cultures, 4 h: *p*_MI432 25 μM_ = 0.0146. Relative absorbance values were calculated by considering the mean absorbance value of control hepatocyte mono-cultures at 4 h as 1 (mean ± SEM; *n*_ctr_ = 6, *n*_MI_ = 3/group; * *p* < 0.05).

The inhibitors MI432 and MI460 did not significantly influence the concentration of MDA at any concentration for any time in the supernatant of cell cultures. In one case, there was a mild increase by MI432 at 25 μM for 4 h on hepatocyte mono-cultures (Figure 7A,B).

**Figure 8:**
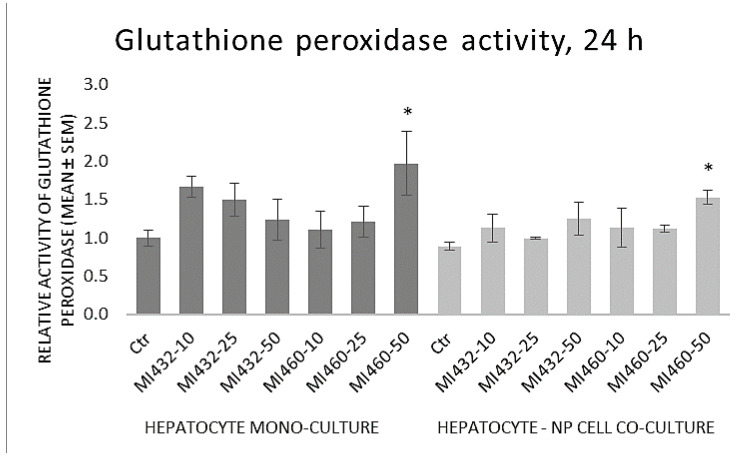
The effects of MI432 and MI460 on the glutathione peroxidase (GPx) activity of hepatocyte mono-cultures and hepatocyte–NP cell co-cultures after 24 h of treatment at 10, 25, and 50 µM, assessed by a specific colorimetric assay kit. *p* values belonging to the significant differences: mono-cultures: *p*_MI460 50μM_ = 0.0446; co-cultures: *p*_MI460 50 μM_ = 0.0273. Relative activity values were calculated by considering the mean of control hepatocyte mono-cultures as 1 (mean ± SEM; *n*_ctr_ = 6, *n*_MI_ = 3/group; * *p* < 0.05).

The GPx activity of cell lysates was significantly increased by the highest concentration (50 μM) of MI460 on both mono- and co-cultures, while no significant differences were observed in the other cases compared to controls (Figure 8).

## 4. Discussion

The present study investigated the effects of matriptase inhibition on cellular metabolic, inflammatory, and redox parameters of hepatic cell culture models, highlighting the potential regulatory role of matriptase in the chicken liver. The applied hepatocyte mono-cultures and hepatocyte–NP cell co-cultures were previously established and characterized by Mackei et al. [23], considered as proper models for studying the avian hepatocellular inflammatory and stress response. Hence, they could serve as good tools for investigations related to the consequences of matriptase inhibition in birds as well.

In the present study, based on the results of the CCK-8 assay, the aerobe catabolic activity of the cultured cells moderately decreased after applying both inhibitors MI432 and MI460 in certain concentrations for 4 h. However, the extent of the reduction indicated that the inhibitors were not cytotoxic, in line with a previous study in which T-2 toxin caused a similar decline in the metabolic activity of the same cell culture models without being cytotoxic [25]. Following the longer, 24 h incubation, no significant differences were observed between control and MI-treated cells anymore, suggesting the rapid metabolic adaptation of liver cells to the applied MIs.

Concerning the extracellular proinflammatory cytokine levels, both IL-6 and IL-8 concentrations of cell-free supernatants were remarkably increased on all MI432-exposed mono- and co-cultures, both after 4 and 24 h incubation time (with the exception of IL-6 for the co-cultures treated with MI432 at 10 µM for 4 h). The MI-triggered elevation in the cytokine release was more pronounced on mono-cultures than on co-cultures after 4 h, but the extent was approximately the same after 24 h exposure on both cell culture models. The more notable MI432-evoked cytokine release of mono-cultures might be related to the finding that hepatocytes can predominantly produce a variety of proinflammatory cytokines, such as IL-6 and IL-8, playing a key role in the inflammatory and stress response [26,27]. Since H_2_O_2_ as a redox signaling molecule can induce hepatocellular IL-8 production [26], the elevated IL-8 levels might have been partly mediated by the increased extracellular ROS concentration in case of MI432 at 25 and 50 μM for 4 on mono-cultures, which was also reflected by the observed positive correlation between H_2_O_2_ and IL-8 levels on mono-cultures. Based on these findings, MI432 might have influenced the crosstalk of different inflammatory mediators, resulting in elevated IL-6 and IL-8 levels, and suggesting the putative role of matriptase in retaining the hepatic oxidative and inflammatory homeostasis via a multifaceted mode of action. In contrast to MI432, no proinflammatory action was exerted by MI460 as indicated by IL-6 and IL-8 concentrations (except IL-8 after 10 µM for 24 h on mono-cultures). Notwithstanding that both MI432 and MI460 are similarly structured 3-APhA based inhibitors, our results confirm that they can possess largely different actions on cell function, possibly due to their different Ki values, particularly for the inhibition of matriptase-2, highly expressed in the liver [17]. Since the inhibition of matriptase with MI432 triggered an intense surge in hepatocellular IL-6 and IL-8 release, our present results suggest that physiologically regulated matriptase may have a key role in maintaining the homeostasis in the liver of chickens, avoiding the excessive production of proinflammatory cytokines. Furthermore, as the elevated interleukin levels of MI432-treated chicken hepatic cell cultures were not alleviated after 24 h incubation, it can be suggested that the fast metabolic adaptation of liver cells to MIs was not coupled to the restoration of the physiological inflammatory homeostasis.

The pleiotropic function of matriptase in the inflammatory response has been studied already in various mammalian cell types, but it was not completely assessed in avian tissues before. The effects of MI432 and MI460 on proinflammatory cytokine production were monitored by Pomothy et al. [17] on similar hepatic cell culture models originated from swine. According to their findings, the short-term (2 h) MI application did not affect the extracellular IL-6 and IL-8 levels of porcine hepatocyte–Kupffer cell co-cultures. Similarly, exposing monolayers and hydrogel-assisted 3D cultures of porcine hepatocyte mono-cultures and hepatocyte–Kupffer cell co-cultures to another 3-APhA type inhibitor, MI461, for 24 h was not capable of altering the IL-6 and IL-8 concentrations of culture media [28]. In humans, matriptase activation was coupled to several inflammatory skin disorders [29], and matriptase induced IL-6 and IL-8 production of endothelial cells via PAR-2-mediated inflammatory signaling [19]. The activation of the PAR-2 pathway is strongly involved in the humoral and cellular inflammatory response promoted by dysregulated matriptase enzymes in context with several diseases, including epidermal tumors [7,30]. However, matriptase possessed a protective role against ulcerative colitis by restoring the gut-barrier function [31]. The observed differences between data gained from mammalian cells and those of the present study using chicken cell cultures highlight the importance of species-dependent differences in matriptase action, and also reflect the complex interplay of matriptase activity and the inflammatory response.

Based on the results obtained, the redox state of the cells was mostly not influenced by the applied inhibitors, since no significant changes were found in extracellular H_2_O_2_ and MDA levels and cellular GPx activity following most of the applied MI treatments. However, in case of MI432 at 25 and 50 µM for 4 h on mono-cultures, a significantly increased ROS level was detected, combined with an elevated lipid peroxidation rate at 25 µM as indicated by the MDA measures. In contrast, 10 µM MI460 showed antioxidant action on co-cultures for both incubation times. The GPx activity of MI460-exposed cells was remarkably elevated only when applying the inhibitor in the highest concentration on both cell culture models. These data suggest that the applied 3-APhA type inhibitors may not strongly affect the hepatic cellular redox homeostasis of chickens in most concentrations; however, they might act as redox modulators under certain conditions. Similarly, the extracellular ROS levels of porcine hepatic cell culture models were not influenced by a short-term (2 h) exposure to 50 µM MI432 and MI460, but the similar 3-AphA type MI441 significantly stimulated the cellular ROS release [17]. On 3D hepatic cell culture models of pig origin, the H_2_O_2_ level was not altered after applying MI461 for 24 h [28]. The stability of the redox homeostasis of matriptase-modulated hepatocytes was also confirmed by our previous study, as sphingosine 1-phosphate as a matriptase inducer did not disturb the oxidative status of primary rat hepatocyte cultures [32]. Concerning the extrahepatic tissues, MI432 caused a transient increase in cellular ROS production of the porcine small intestinal cell line IPEC-J2 when applied for 2 h at 50 µM, but it was normalized during longer exposure times [22]. In contrast, the exposure of IPEC-J2 monolayers to MI439 and MI476 for 24 h elicited a remarkable antioxidative action by decreasing the extracellular H_2_O_2_ concentration on IPEC-J2 monolayers, which effect was the most pronounced at the lowest applied concentration (10 μM) [33], similarly to the action of MI460 on hepatic co-cultures in the present study. Concluding these data, matriptase inhibition might slightly influence the ROS production and the cellular antioxidative defense mechanisms in certain cases, but it cannot be considered as a major modulator of the cellular redox homeostasis. The MI460-driven activation of GPx as a major antioxidant system can be suggested to alleviate the cellular ROS release, preventing hepatic oxidative distress and contributing to the absence of increased ROS and MDA levels. Hence, addressing the results of the present study, it can be suggested that MIs do not greatly affect the oxidative state of cultured chicken liver cells, and thus they might be applied without causing oxidative distress and lipid peroxidation.

Comparing the MI-driven changes on mono- and co-cultures, no considerable differences could be found, similar to the study of Pászti-Gere et al. [28] involving porcine hepatocyte mono-cultures and hepatocyte–Kupffer cell co-cultures. This finding may suggest that the presence of hepatic NP cells is not a critical factor in determining the effects of matriptase inhibition. As the presently applied co-culture with a cell ratio of 6:1 (hepatocyte to NP cells) served as a model of hepatic inflammation with moderate macrophage infiltration [23], it can be suggested that 3-AphA based MIs may act similarly under physiological and mildly inflamed conditions on the cells of the chicken liver. However, the proinflammatory action of MI432 on both cell culture models highlights that matriptase may be considered as a key regulator of hepatic inflammation in chicken, preventing excessive proinflammatory cytokine release. Notwithstanding that the underlying mechanisms of the aforementioned effects should be investigated in further studies, the present trial provided the first evidence regarding the role of matriptase in maintaining physiological homeostasis of the avian liver. If matriptase inhibitors were considered to be applied as drug candidates in the future in veterinary medicine, this newly suggested function of matriptase in avian species should be carefully addressed.

## 5. Conclusions

In conclusion, the present study revealed that matriptase inhibition by MI432 triggered intense and substantive proinflammatory IL-6 and IL-8 production in chicken-derived hepatic cell culture models, which was not accompanied with enhanced oxidative stress and lipid peroxidation as indicated by cellular ROS production and MDA levels. The application of both inhibitors MI432 and MI460 was not cytotoxic, but caused a transient moderate metabolic depression of cultured liver cells following 4 h exposure, which was restored after 24 h, reflecting the fast hepatic adaptation potential to MI administration. These results may suggest that physiological matriptase activities should have a key function in retaining the metabolic and inflammatory homeostasis of the liver in chicken, without being a major modulator of the hepatocellular redox state.

## Figures and Tables

**Table 1:** The K_i_ (inhibitory constant) values of the applied matriptase inhibitors.

Ki (µM)	Matriptase-1	Matriptase-2
MI432	0.002	0.11
MI460	0.0018	0.18

## Data Availability

All raw data supporting the results of the present study can be obtained from the corresponding author upon reasonable request.
